# Antibacterial Performance of Terpenoids from the Australian Plant *Eremophila lucida*

**DOI:** 10.3390/antibiotics8020063

**Published:** 2019-05-17

**Authors:** Israt J. Biva, Chi P. Ndi, Susan J. Semple, Hans J. Griesser

**Affiliations:** 1 Future Industries Institute, University of South Australia, Mawson Lakes 5095, Australia; israt.biva@mymail.unisa.edu.au; 2 Wound Management Innovation Cooperative Research Centre, Toowong 4066, Australia; 3 Quality Use of Medicines and Pharmacy Research Centre, School of Pharmacy and Medical Sciences, University of South Australia, Frome Road, Adelaide 5000, Australia; chi.ndi@unisa.edu.au (C.P.N.); susan.semple@unisa.edu.au (S.J.S.)

**Keywords:** *Eremophila*, Scrophulariaceae, diterpenoid, sesquiterpenoid, antimicrobial, antibacterial

## Abstract

Plants in the Australian genus *Eremophila* (Scrophulariaceae) have attracted considerable recent attention for their antimicrobial compounds, which possess a wide range of chemical structures. As they are typically associated with the oily-waxy resin layer covering leaves and green branchlets, and *Eremophila lucida* is prominent among the species containing a pronounced sticky resin layer, this species was considered of interest for assessing its antibacterial constituents. The *n*-hexane fraction of the crude acetone extract of the leaves exhibited antibacterial activity against *Staphylococcus aureus*. Isolation led to the known compounds cembratriene, (3*Z*, 7*E*, 11*Z*)-15-hydroxycembra-3,7,11-trien-19-oic acid (1), the sesquiterpenoid, farnesal (2) and the viscidane diterpenoid, 5α-hydroxyviscida-3,14-dien-20-oic acid (3). The purified compounds were tested for antibacterial activity with 2 and 3 showing moderate antibacterial activity against Gram-positive bacteria.

## 1. Introduction

There is continuing scientific interest in elucidating the scientific basis for traditional usage of medicinal plants and the potential for identification of new lead antimicrobial compounds. Among the medicinal plants used in Australian Aboriginal cultural traditions, species from the genus *Eremophila* R.Br. (Scrophulariaceae) figure prominently. This genus is endemic to Australia and concentrated mainly in arid and semi-arid regions, as suggested by its generic name deriving from the Greek *Eremos*, desert, and *phileo*, to love. A large genus with species varying considerably in appearance, the taxonomy of *Eremophila* was reviewed in detail by Chinnock [1] showing that the genus comprises over 218 species, with a number still undescribed. 

Phytochemical investigations of species in the genus *Eremophila* have led to the isolation and identification of over 200 secondary compounds from several classes, with particularly rich diversity in sesqui- and diterpenoids [2]. This diversity of secondary compounds and the traditional use of some species has highlighted the pharmacological potential of this genus [3,4,5]. In more recent years biological activities have been found for various *Eremophila* extracts and isolated secondary compounds, including anti-inflammatory, antimicrobial and cytotoxic activities, and cardioactive effects [2,6,7,8,9,10,11]. The compounds reported to be bioactive are mostly serrulatane diterpenes and flavonoids.

A screening survey showed that antimicrobial compounds typically are found in species that contain a sticky, oily or waxy layer of resin on leaves and green branchlets [9]. Hence, *Eremophila* species producing substantial amounts of resin would seem to be candidates for detailed study of isolation and antimicrobial performance of bioactive compounds. One such plant species is *Eremophila lucida* Chinnock; it is commonly known as ‘shining poverty bush’ as the leaves are very shiny and viscid in nature, indicating the presence of large quantities of resin. This species grows as an erect and glabrous shrub [1]. It is not known from traditional lore to have been used medicinally, but this could be due to its very restricted natural distribution in Western Australia in an area where traditional culture has been impacted markedly. Previously, as part of a study of various *Eremophila* species, we found that an extract of the leaves of *E. lucida* exhibited antibacterial activity against Gram-positive bacteria including *Staphylococcus* and *Streptococcus* species [12]; however, the chemical constituents were not isolated in that survey work. As a continuation of our research to examine antimicrobial compounds in *Eremophila* species, here we isolated three major constituents of *E. lucida* leaf resin from the *n*-hexane fraction of the initial crude extract, and examined their in vitro antibacterial activity.

## 2. Experimental Section

### 2.1. Methods and Instruments

The 1D and 2D NMR data were acquired on a Varian INOVA 600 MHz spectrometer in CD_3_OD or CDCl_3_ (Sigma-Aldrich, St Louis, MO, USA). Chemical shifts in the NMR spectra were assigned by reference to the signals from the residual solvents. Mass spectra were recorded on a Triple ToF 5600 ABC Science mass spectrometer. Low pressure column chromatography used Sephadex LH-20 (Sigma-Aldrich), a 270 × 45 mm glass chromatographic column, silica gel (60 Å pore size, Merck, Darmstadt, Germany), and a 550 × 27 mm glass column. Further separations were performed through HPLC, consisting of a two-pump LC-8A unit (Shimadzu, Japan), with a UV/vis detector SPD-20A (Shimadzu), a communication bus module CBM-20A (Shimadzu), fraction collector FRC-10A (Shimadzu), software LC Solution (Shimadzu), and a C18 reverse phase (semi-preparative) column 300.0 × 7.8 mm, 125Å, 5 μm (Waters, USA) or analytical column (250 × 4.60 mm, 3 μm). Silica-gel 60 F_254_ aluminium plates (Merck), were used for thin layer chromatography (TLC) to detect compounds of interest. All solvents used for extractions from plant materials and separations were analytical or HPLC grade (Merck and Univar, Ajax Finechem, Auckland, New Zealand). Formic acid and sulfuric acid were reagent grade (Scharlau, Australia) and glacial acetic acid was analytical grade (Chem Supply, Australia).

### 2.2. Plant Material

Due to the Conservation Code rating of this species, collection from the wild was not feasible. Leaves of *E. lucida* grown in cultivation in a private garden were collected near Gumeracha, South Australia (GPS coordinates: 34.8326 S, 138.8928 E, elevation 360 m) in April 2012. The source was a cultivated plant which was morphologically identical to wild collections and grown in loamy soil under similar conditions though with slightly higher annual rainfall. A voucher specimen (AD191408) was deposited at the State Herbarium of South Australia, Adelaide, South Australia and its identity confirmed by *Eremophila* taxonomist Dr. Robert J Chinnock.

### 2.3. Extraction and Isolation

Fresh entire leaves (122 g) of *E. lucida* were soaked in acetone overnight at room temperature in a closed conical glass container (1 L). The solvent was decanted and evaporated in vacuo to dryness (55 °C) to provide a crude extract (22 g). The whole extract was dissolved in 200 mL MeOH-H_2_O (7:3) and exhaustively partitioned with solvents, 100% *n*-hexane (4 × 200 mL) and then with 100% CH_2_Cl_2_ (4 × 250 mL), to yield the *n*-hexane (9.5 g), CH_2_Cl_2_ (9 g) and the aq. MeOH fractions, respectively.

Of these three fractions of the initial crude extract, the *n*-hexane fraction (9.5 g) showed the strongest antibacterial activity and thus was selected for detailed investigation; it was subjected to Sephadex LH-20 CC using a mobile phase of CH_2_Cl_2_-MeOH (3:1). An initial fraction of 100 mL was collected, and then 35 × 5 mL fractions were collected. Based on their TLC profile, fractions were pooled into three major groups (F1-8, F9-24, and F25-35). Of these, the fraction F9-24 (6 g dried) showed the strongest antibacterial activity and thus was selected for further separation; it was re-dissolved in 20% *v/v* CH_2_Cl_2_ in *n*-hexane and further partitioned sequentially first with a 8% *w/v* NaHCO_3_ solution (2 × 100 mL), and then with a 5% *w/v* NaOH solution (2 × 100 mL). This resulted to three portions, the 8% NaHCO_3_ portion, the 5% NaOH portion, and the CH_2_Cl_2_- *n*-hexane portion, respectively. After acidification with conc. H_2_SO_4_, the basic portions 8% NaHCO_3_ and 5% NaOH were extracted with CH_2_Cl_2_ (3 × 100 mL) to yield the NaHCO_3_-soluble, the NaOH-soluble, and the neutral CH_2_Cl_2_ fractions. All fractions were dried in vacuo.

The NaHCO_3_-soluble fraction (1 g) was subjected to silica gel column chromatography. Step gradient elution was conducted by using the mobile phase *n*-hexane-EtOAc (100% *n*-hexane through to 100% EtOAc + 0.1% *v/v* HCOOH) to give 96 fractions which were grouped into eight fractions based on their TLC profile (F1-10, F11-19, F20-24, F25-30, F31-36, F37-55, F56-75 and F76-96). Further separation of pooled fractions F11-19, F37-55, F56-75 and F76-96 was conducted by HPLC.

Fraction F76-96 (90 mg) was separated through preparative RP-HPLC using the Waters C18 semi-preparative column and an isocratic mobile phase of MeOH-H_2_O (3:1 with 0.1% HCOOH), with a flow rate of 2 mL/min, collecting 32 × 2 mL fractions in three separate runs (30 mg/run). The HPLC fractions 4–6 yielded the semi-pure compound **1** while fractions 7–9 yielded pure compound **1** (3 mg) as an amorphous white solid. The HPLC fractions 4–6 (20 mg) were subjected to further reverse phase HPLC using an analytical column and an isocratic mobile phase of MeOH-H_2_O (4:1 with 0.1% HCOOH), a flow rate of 0.5 mL/min, collecting 45 × 1 mL fractions in 20 separate run (1 mg/run) to yield a further 8 mg of 1.

Fraction F11-19 (105 mg) from silica gel CC was further separated through reverse phase HPLC using a preparative column and an isocratic mobile phase of MeOH: H_2_O (4:1 + 0.1% HCOOH), and a flow rate of 2 mL/min, collecting 60 × 2 mL fractions in three separate runs (30 mg/run). Fractions 20–25 gave semi-pure fractions (36 mg, containing compound **2**). These HPLC fractions (24 mg) were further subjected to RP HPLC using an analytical column and an isocratic mobile phase of MeOH: H_2_O (4:1 with 0.1% CH_3_COOH), flow rate 0.8 mL/min, collecting 30 × 1 mL fractions. The separation was repeated 24 times (1 mg/run) to separate sufficient quantities of the pure compound. Fractions 9–10 yielded the pure compound **2** (7 mg) as a white powder.

The fractions F37–55 (25 mg) and F56–75 (60 mg) from silica gel CC were further separated through RP-HPLC using a preparative column and an isocratic mobile phase of MeOH: H_2_O (3:1 + 0.1% HCOOH) with a flow rate of 2 mL/min, collecting 60 × 2 mL fractions. Sub-fractions 30–35 and 32–38 from F37–55 and F56–75, respectively, yielded the pure compound **3** (10 mg) as a white amorphous solid.

### 2.4. Antibacterial Assays

As in our previous work with other *Eremophila* plant species [11,13,14], the crude extract, successive fractions, and isolated pure compounds were tested against the Gram-positive and Gram-negative bacterial strains *Staphylococcus aureus* ATCC 29213, *S. aureus* ATCC 25923 and *Escherichia coli* ATCC 25922. These bacteria were obtained from stock cultures preserved at −80 °C at the School of Pharmacy and Medical Sciences, University of South Australia. Bacteria were grown on blood agar plates (Colombia agar CM 331, Oxoid, England; supplemented with 5% *v/v* sheep blood) at 37 °C. Cation adjusted Mueller Hinton (MH) II broth (Becton Dickinson, France) was used for experiments to determine the minimum inhibitory concentration (MIC) and the minimum bactericidal concentration (MBC) for the crude extract and the pure compounds.

To determine the MIC a broth microdilution technique was used [13]. Duplicate 2-fold serial dilutions of test samples were prepared in sterile round bottom 96-well plates (Sarstedt, Technology Park, Australia) in MH broth containing 2% *v/v* DMSO. Bacterial cell suspension (100 μL) corresponding to 1 × 10^6^ CFU/mL was added to wells; other wells were used for saline, test sample sterility, and media sterility controls, respectively. The final concentration of bacteria was 5 × 10^5^ CFU/mL and that of DMSO was 1% *v/v*. This concentration of DMSO did not affect bacterial growth. After shaking for 10 min, plates were incubated overnight at 37 °C. The MIC was determined as the lowest concentration at which no growth was observed in duplicate wells. Ampicillin and gentamicin (Sigma) were used as positive controls for the Gram-positive and the Gram-negative bacteria, respectively. Following the determination of the MIC, the MBC was determined [13] by transferring a 10 μL aliquot from each well at the concentrations corresponding to the MIC and above, and adding 190 μL of MH broth in a separate sterile 96-well plate. Plates were incubated under the same conditions as that described for MIC tests. The presence or absence of bacterial growth was determined by visual inspection. The MBC was determined as the lowest concentration in the original plate at which no growth was seen in the second plate.

## 3. Results

**Figure 1:**
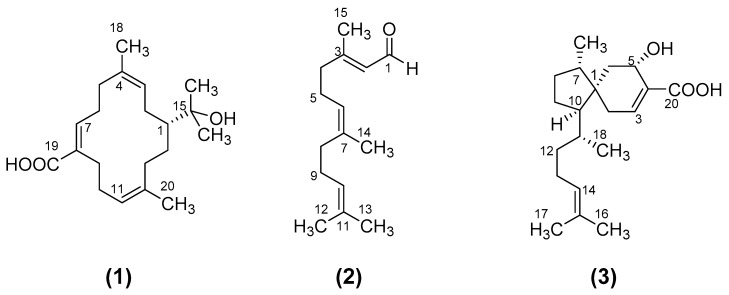
Compounds isolated in this study from *Eremophila lucida.*

The chemical structures of the three compounds isolated from *E. lucida* and tested for antibacterial activity are shown in Figure 1.

The structures of the compounds were identified by detailed analysis of mass spectra and 1D/2D NMR spectra (^1^H and ^13^C NMR spectra, ^1^H-^1^H COSY, HSQC and HMBC) and by comparisons of their spectroscopic data with those reported in the literature. The 1D and 2D NMR data for the compounds are provided in the Supporting Information (Figures S1–S17). Compound **1** was identified as the cembrene diterpenoid, (3*Z*, 7*E*, 11*Z*)-15-hydroxycembra-3,7,11-trien-19-oic acid which has previously been isolated from *E. lucida* [15] and other *Eremophila* species [16]. Compound **2** was identified as the known sesquiterpene farnesal ((2*E*, 6*E*)-3,7,11-trimethyl-2,6,10-dodecatrienal) [17]. Compound **3** was identified as the known viscidane diterpenoid 5α-hydroxyviscida-3,14-dien-20-oic acid [18,19]. This compound had previously been isolated from *E. lucida* [15] and *Eremophila viscida* [18], however it has not previously been investigated for antimicrobial activity.

The acetone crude extract of *E. lucida* leaves and the *n*-hexane fraction from this crude extract were found to possess antimicrobial activity with MIC values of 250–500 μg/mL and 250–300 μg/mL, respectively, against the bacterial strains *Staphylococcus aureus* ATCC 29213 and *S. aureus* ATCC 25923, but did not show activity against *E. coli*. The pure compounds **1**–**3** were tested for their antibacterial activity. Compound **2** showed modest antibacterial activity against Gram-positive bacterial strains *S. aureus* ATCC 29213 and *S. aureus* ATCC 25923 with a MIC of 65 μg/mL (295 μM). Compound **3** was found to be active against *S. aureus* ATCC 25923 with an MIC of 62.5 μg/mL (195 μM) but did not show activity against *S. aureus* ATCC 29213 at the maximum concentration of 125 μg/mL tested. The cembrene 1 was not active against the same strains at the maximum concentration tested. No activity was observed for these compounds against the Gram-negative bacterial strain *E. coli* ATCC 25922 up to a maximum test concentration of 125–130 μg/mL. The positive controls ampicillin and gentamicin gave MIC values of 0.6 μg/mL (0.2 μM) and 1.0 μg/mL (0.4 μM) for the Gram-positive and the Gram-negative bacteria, respectively.

## 4. Discussion

The three compounds we have isolated from the *n*-hexane fraction of the crude extract include a cembratriene and a viscidane diterpenoid (1 and 3) and a sesquiterpenoid (2). A number of *Eremophila* species have been previously found to produce macrocyclic and bicyclic groups of diterpenes that are based on the unique cembrane skeleton (containing an internal cis double bond) or on the viscidane skeleton [20,21]. NMR analyses indicated that compound 2 was the sesquiterpene farnesal, a compound that was previously found in plants of the genus *Leptospermum* as one of the components of essential oils [22].

Compounds **2** and **3** showed antibacterial activity at similar concentrations against the bacterial strain *S. aureus* 25923; however, compound **2** also showed activity (MIC 65 μg/mL) against the other strain *S. aureus* ATCC 29213. In contrast compound **1** did not show any activity against the same bacterial strains.

Farnesal and the structurally related compound farnesol have previously been reported to have antimicrobial activity [23]. Farnesol exerts antibacterial activity by disrupting the cell membrane and it was also found that it can destroy biofilms of Gram-positive bacteria by reducing biomass [24,25]. Although the mechanisms responsible for antibacterial activity of farnesal have not yet been reported, it appears reasonable to hypothesise that farnesal would act in the same way as farnesol, possibly by its hydrophobic nature facilitating insertion into the bacterial phospholipid bilayer membrane and consequent structural disruption.

Although viscidane diterpenes are common in the genus *Eremophila* [2], their activity against bacteria has not been evaluated to date. The antibacterial activity we observed of the bicyclic viscidane diterpenoid 3 against Gram-positive bacteria might be related to membrane insertion, given the similarity of an isoprene tail in its structure with those of farnesal, farnesol, several antibacterial serrulatane diterpenoids isolated from other *Eremophila* species [6,12,26], and neryl ferulate and neryl *p*-coumarate recently isolated as antimicrobial components of *Eremophila longifolia* [7]. The hydrophilic tail in the serrulatane structure appears to play a role in antimicrobial activity against the Gram-positive bacterium *S. aureus*, with the previously identified antibacterial serrulatane 8-hydroxyserrulat-14-en-19-oic acid acting by inhibiting bacterial biosynthesis and by membranolysis [27].

The genus *Eremophila* contains a number of unusual and unique cembrene diterpenes, the structures of which possess double bonds in the *cis* configuration [21]. However, no report has been found regarding antibacterial or other pharmacological activity of *Eremophila* cembrenes. The cembratriene 1 was examined for activity against both Gram-positive and Gram-negative bacteria, but it was found not to be active at the tested concentrations. In general, with a diversity of structural variations, cembranoids isolated from other natural sources exhibit various biological activities that include anti-inflammatory, anti-parasitic and cytotoxic properties [28]. Some cembrane diterpenes from soft corals have been tested for activities including antibacterial, anti-inflammatory and anticancer activity, with potent anti-inflammatory activity reported [29,30]. In future work it would be of interest to study such other pharmacological activities of this compound as well as to examine the antibacterial activity of a wider range of cembranoid diterpenoids from *Eremophila* species.

While *Eremophila lucida* possesses large amounts of leaf resin and thus appeared promising, the major constituents we have isolated showed only rather moderate antibacterial activity. This accords with the limited activity of the initial fractions; of the three fractions into which the initial crude extract was separated, the *n-*hexane fraction showed the highest activity but at 250–300 µg/ml the antibacterial activity was inferior to that of other initial fractions from crude extracts [11,13,14]. Perhaps this is why this plant species is not recorded as a traditional medicinal plant, although other possible reasons such as its limited distribution or extinction of local knowledge cannot be excluded. As compounds in the other fractions from the crude extract (dichloromethane, DCM, and methanol, respectively, with the latter not showing any antibacterial activity) were not isolated, we cannot exclude the possibility that *E. lucida* also contains serrulatanes, which seem to be ubiquitous in Eremophilas, but any such compounds seem not to be highly active or the DCM fraction should have shown higher activity.

## 5. Conclusions

In conclusion, the sesquiterpene farnesal and a viscidane diterpene, 5α-hydroxyviscida-3,14-dien-20-oic acid, have been found to contribute to the antibacterial activity of *E. lucida* extracts.

## Supplementary Materials

The following are available online at https://www.mdpi.com/2079-6382/8/2/63/s1, Figure S1: Eremophila lucida, flowering twigs and growth habit underneath Eucalyptus trees, Figure S2: 1H NMR spectrum of compound **1** in CDCl3, Figure S3: 13C NMR spectrum of compound **1** in in CDCl3, Figure S4: DEPT NMR spectrum of compound **1** in CDCl3, Figure S5: 1H-1H COSY NMR spectrum of compound **1** in CDCl3, Figure S6: HSQC NMR spectrum of compound **1** in CDCl3, Figure S7: HMBC NMR spectrum of compound **1** in CDCl3, Figure S8: 1H NMR spectrum of compound **2** in CD3OD, Figure S9: 13C NMR spectrum of compound **2** in in CD3OD, Figure S10: 1H-1H COSY NMR spectrum of compound **2** in CD3OD, Figure S11: HSQC NMR spectrum of compound **2** in CD3OD, Figure S12: HMBC NMR spectrum of compound **2** in CD3OD, Figure S13: 1H NMR spectrum of compound **3** in CDCl3. Figure S14: 13C NMR spectrum of compound **3** in in CDCl3, Figure S15: 1H-1H COSY NMR spectrum of compound **3** in CDCl3, Figure S16: HSQC NMR spectrum of compound **3** in CDCl3. Figure S17: HMBC NMR spectrum of compound **3** in CDCl3.
