# Supplementary material for: Antibacterial Performance of Terpenoids from the Australian Plant Eremophila lucida
Source: Antibiotics (Basel). 2019 May 17;8(2):63. doi: 10.3390/antibiotics8020063 (PMC6627632; doi:10.3390/antibiotics8020063)

Supporting Information

Antibacterial performance of terpenoids from the Australian plant *Eremophila lucida*

Israt J. Biva [a,b], Chi P. Ndi [c], Susan J. Semple [c], Hans J. Griesser [a,b,*],

[a] Future Industries Institute, University of South Australia, Mawson Lakes, SA 5095, Australia

[b] Wound Management Innovation Cooperative Research Centre, Toowong, QLD 4066, Australia

[c] Quality Use of Medicines and Pharmacy Research Centre, School of Pharmacy and Medical Sciences, University of South Australia, Frome Road, Adelaide, SA 5000, Australia

*To whom correspondence should be addressed: Prof. Hans Griesser +61-8-83023703 hans.griesser@unisa.edu.au

List of Supporting Information

Figure S1. *Eremophila lucida*, flowering twigs and growth habit underneath Eucalyptus trees.

Figure S2. ^{1}H NMR spectrum of compound **1** in $CDCl_3$

Figure S3. ^{13}C NMR spectrum of compound **1** in in $CDCl_3$

Figure S4. DEPT NMR spectrum of compound **1** in $CDCl_3$

Figure S5. ^{1}H-^{1}H COSY NMR spectrum of compound **1** in $CDCl_3$

Figure S6. HSQC NMR spectrum of compound **1** in $CDCl_3$

Figure S7. HMBC NMR spectrum of compound **1** in $CDCl_3$

Figure S8. ^{1}H NMR spectrum of compound **2** in CD_3OD

Figure S9. ^{13}C NMR spectrum of compound **2** in in CD_3OD

Figure S10. ^{1}H-^{1}H COSY NMR spectrum of compound **2** in CD_3OD

Figure S11. HSQC NMR spectrum of compound **2** in CD_3OD

Figure S12. HMBC NMR spectrum of compound **2** in CD_3OD

Figure S13. ^{1}H NMR spectrum of compound **3** in $CDCl_3$

Figure S14. ^{13}C NMR spectrum of compound **3** in in $CDCl_3$

Figure S15. ^{1}H-^{1}H COSY NMR spectrum of compound **3** in $CDCl_3$

Figure S16. HSQC NMR spectrum of compound **3** in $CDCl_3$

Figure S17. HMBC NMR spectrum of compound **3** in $CDCl_3$

Figure S1. *Eremophila lucida*, flowering twigs and growth habit underneath Eucalyptus trees. From https://florabase.dpaw.wa.gov.au/browse/profile/17549 (viewed 31/3/2019).

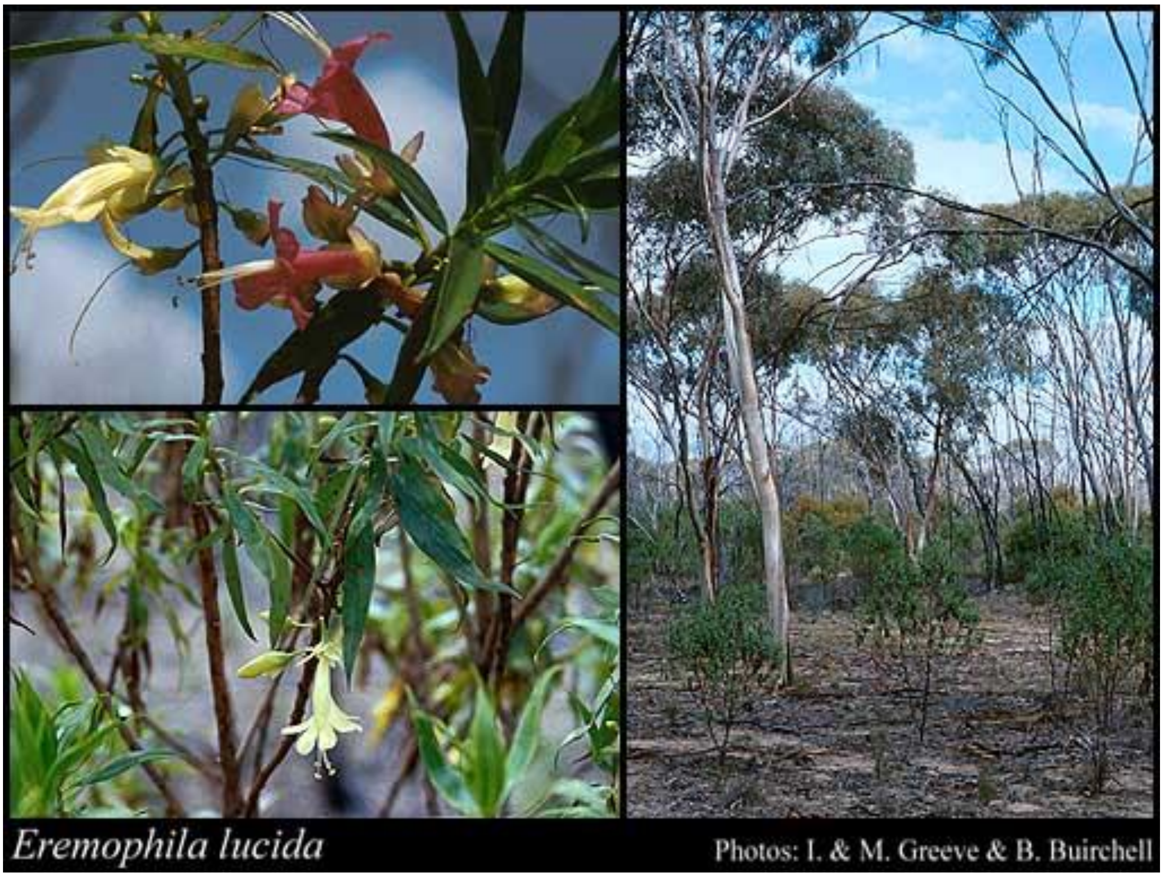

Figure S2. ^{1}H NMR spectrum of compound 1 in $CDCl_3$ (600 MHz)

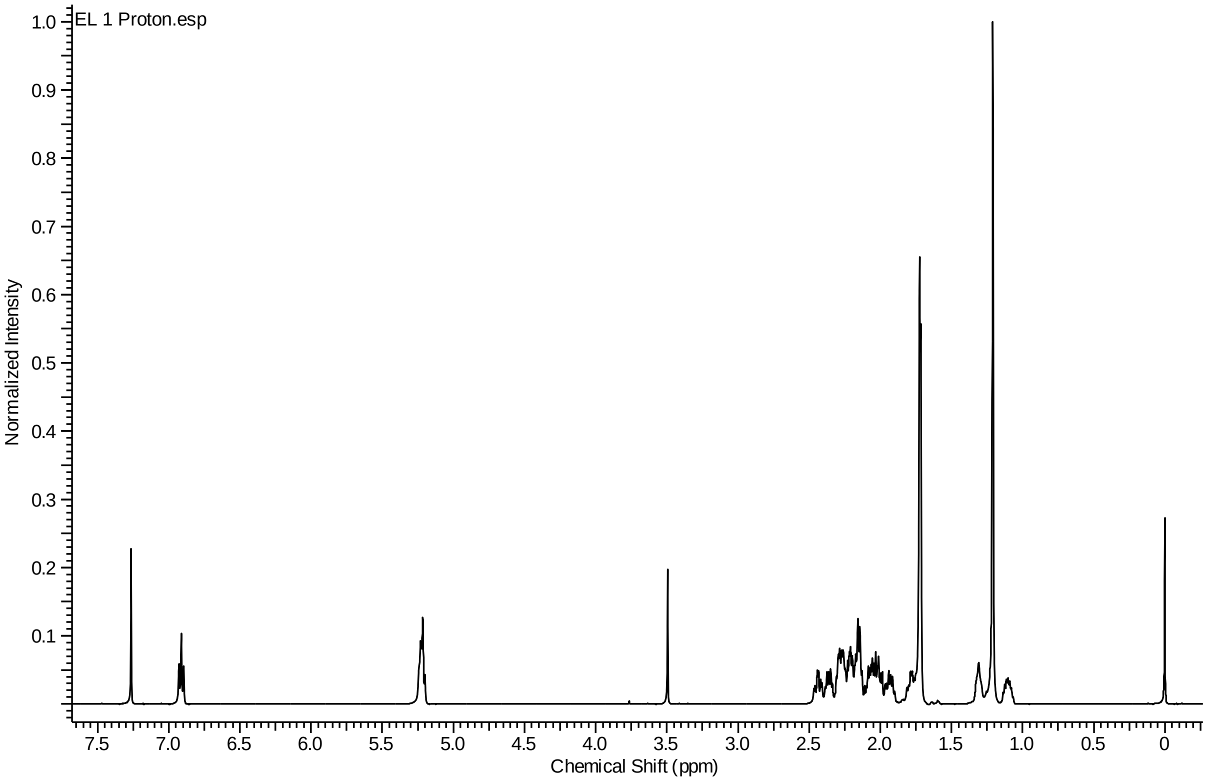

Figure S3. ^{13}C NMR spectrum of compound **1** in in $CDCl_3$

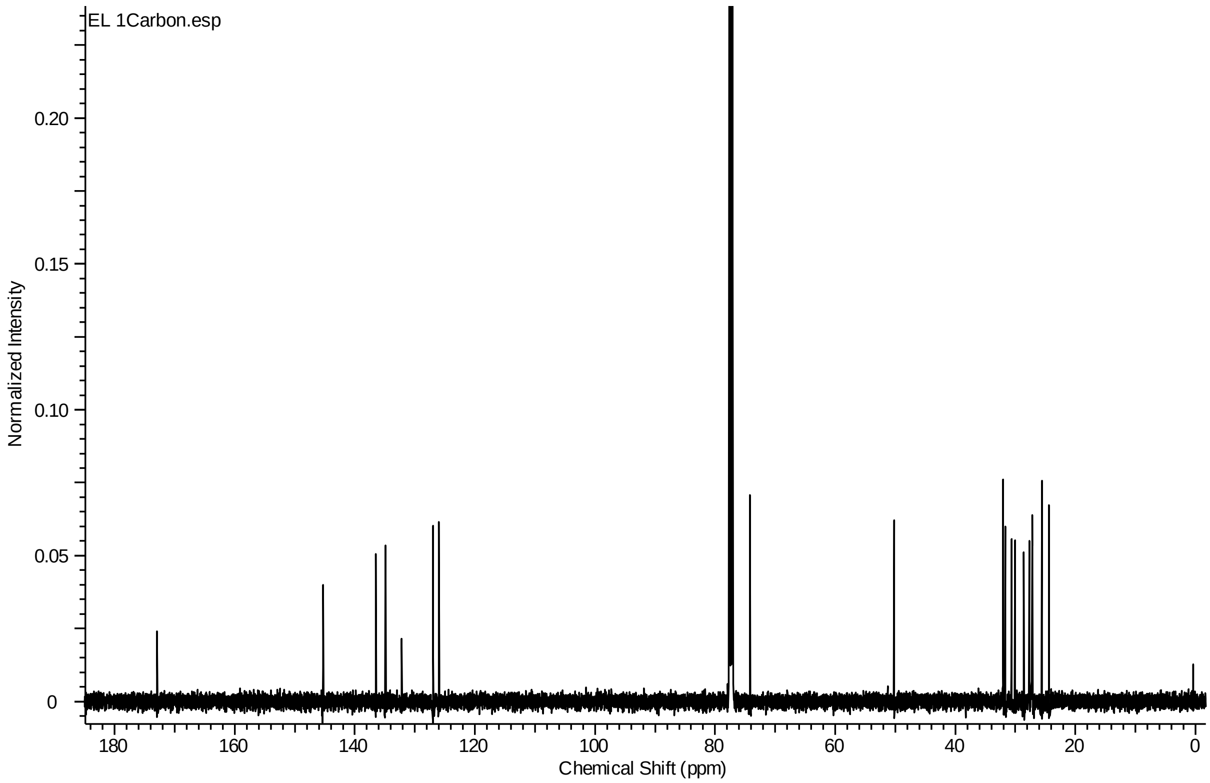

Figure S4. DEPT NMR spectrum of compound **1** in $CDCl_3$

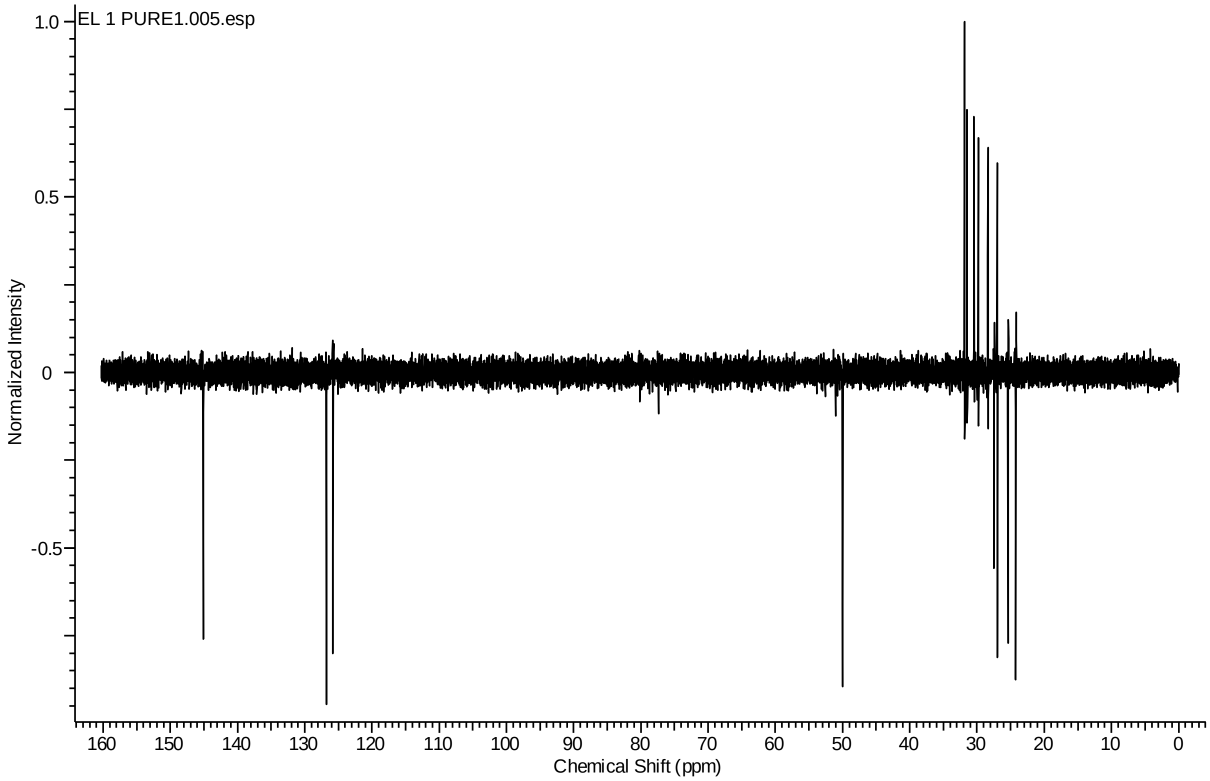

Figure S5. ^{1}H-^{1}H COSY NMR spectrum of compound **1** in $CDCl_3$

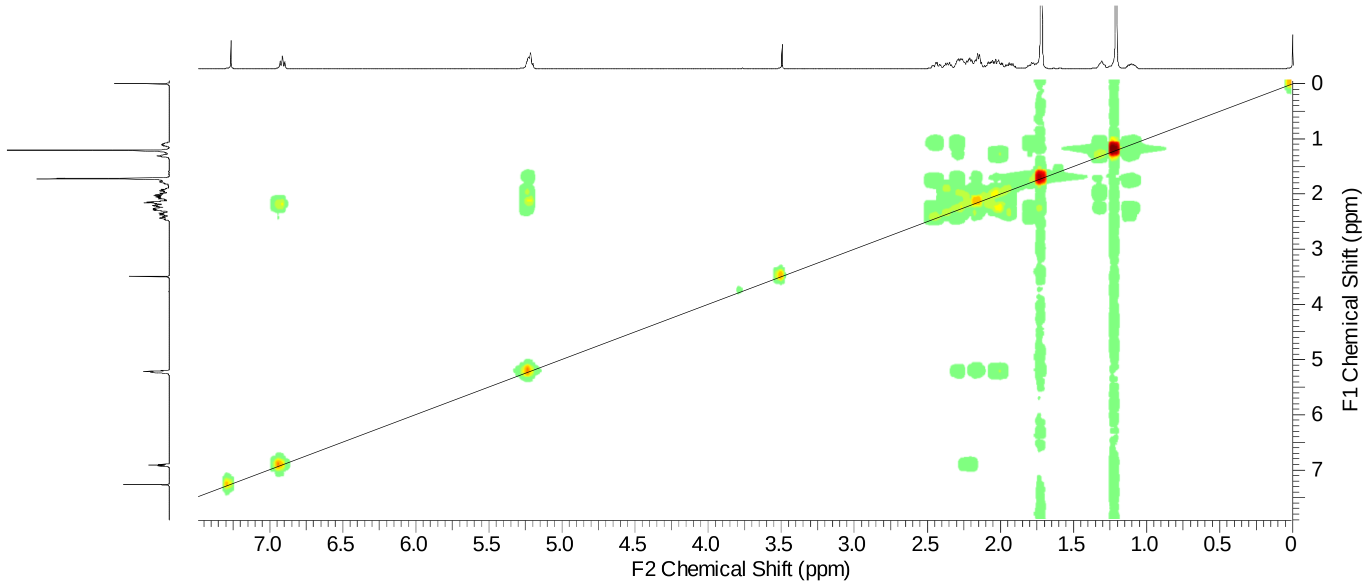

Figure S6. HSQC NMR spectrum of compound **1** in $CDCl_3$

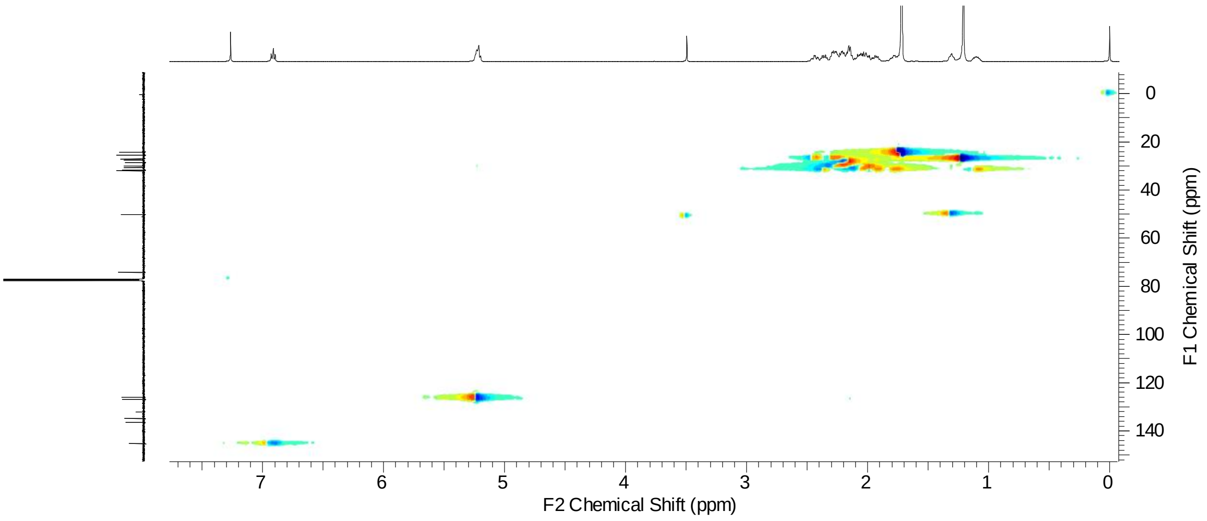

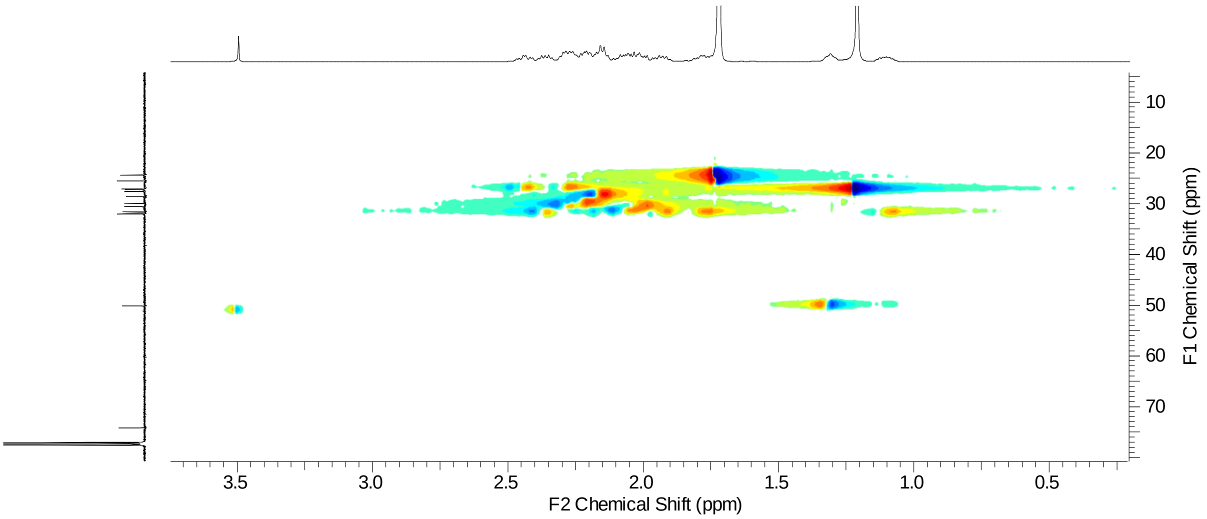

Figure S7. HMBC NMR spectrum of compound **1** in $CDCl_3$

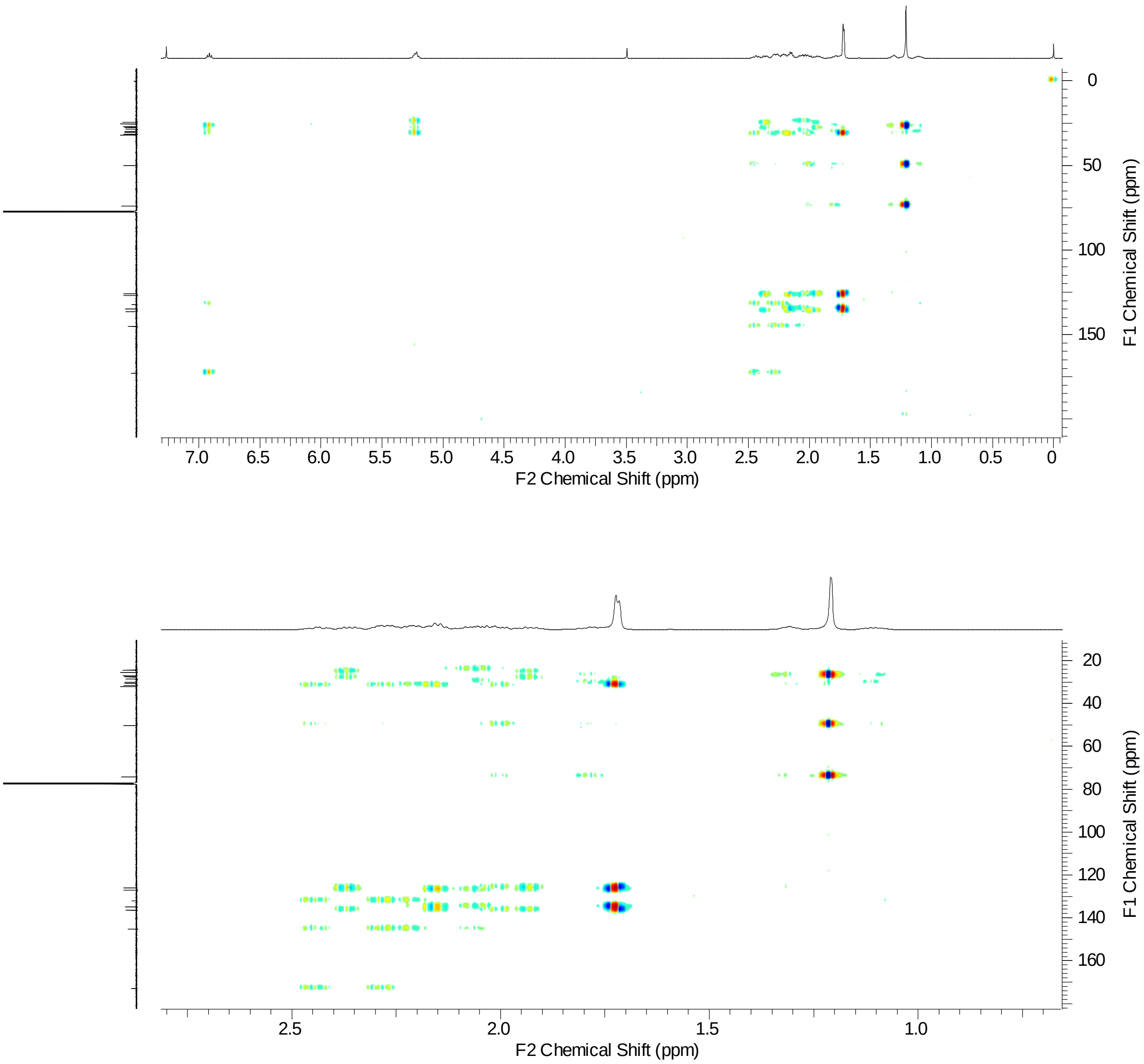

Figure S8. ^{1}H NMR spectrum of compound **2** in CD_3OD (600 MHz)

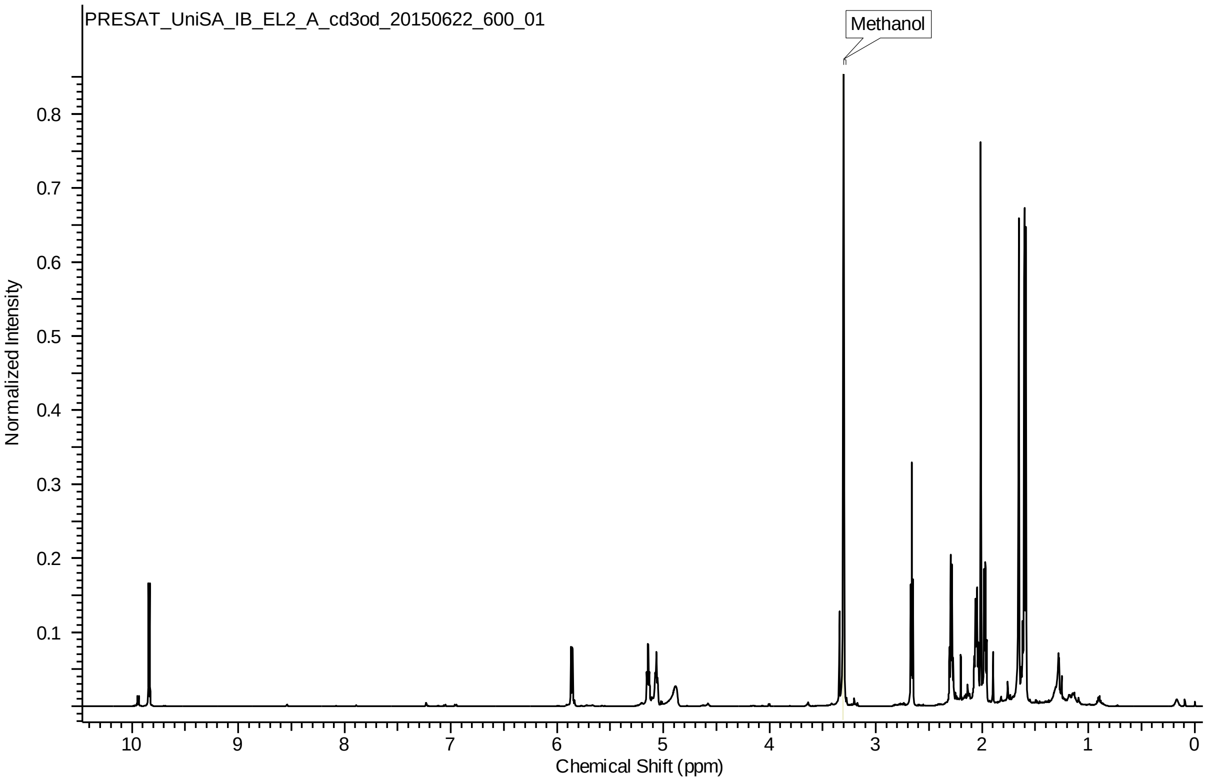

Figure S9. ^{13}C NMR spectrum of compound **2** in CD_3OD

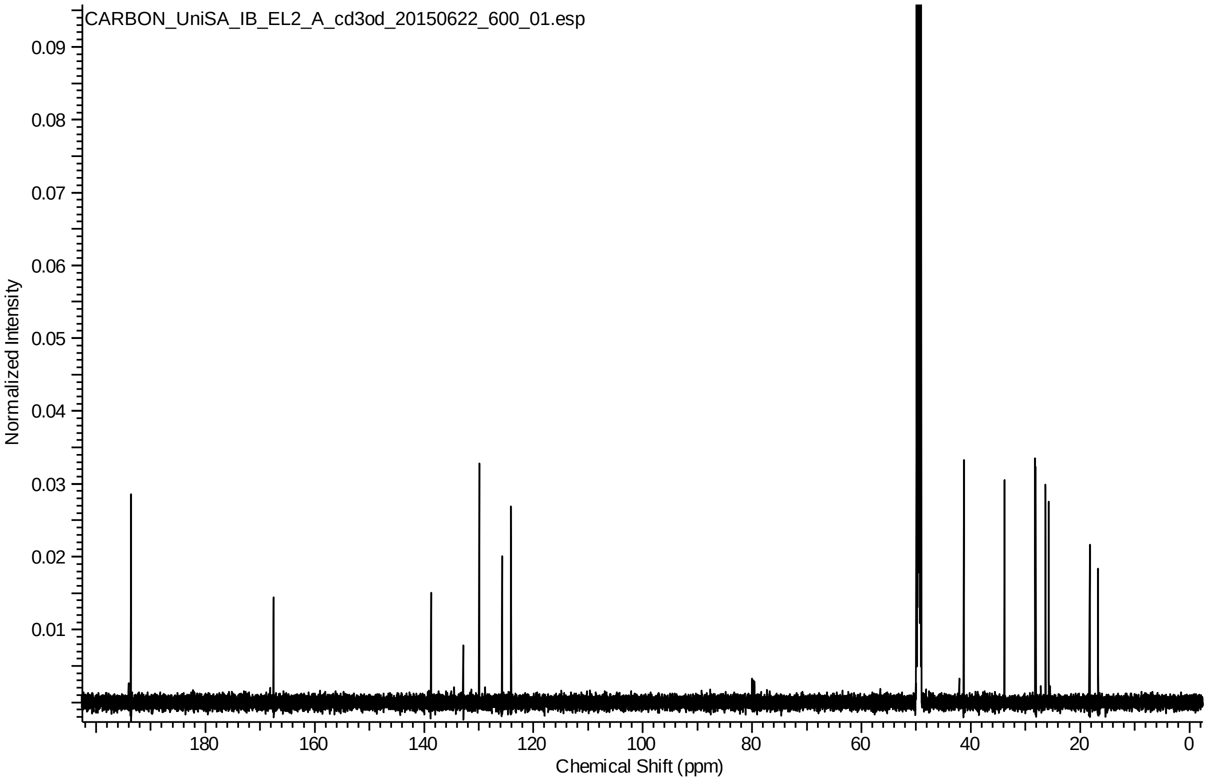

Figure S10. ^{1}H-^{1}H COSY NMR spectrum of compound **2** in CD_3OD

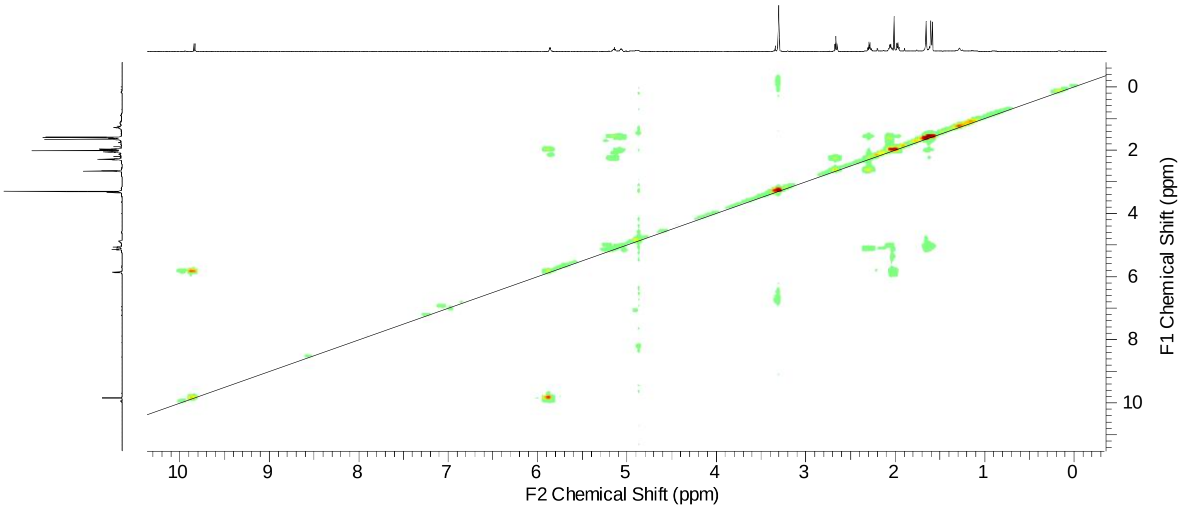

Figure S11. HSQC NMR spectrum of compound **2** in CD_3OD

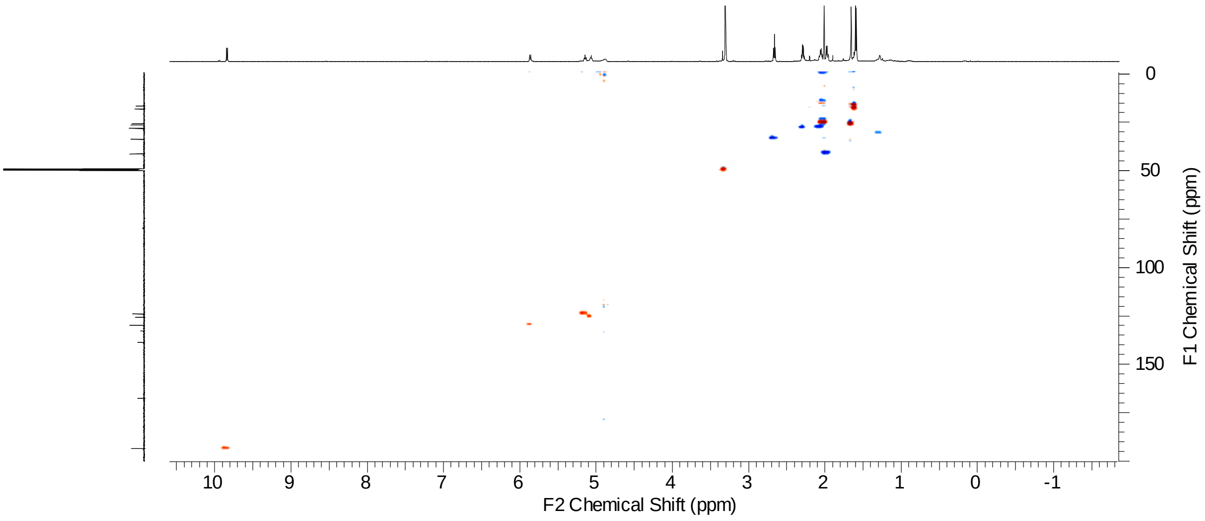

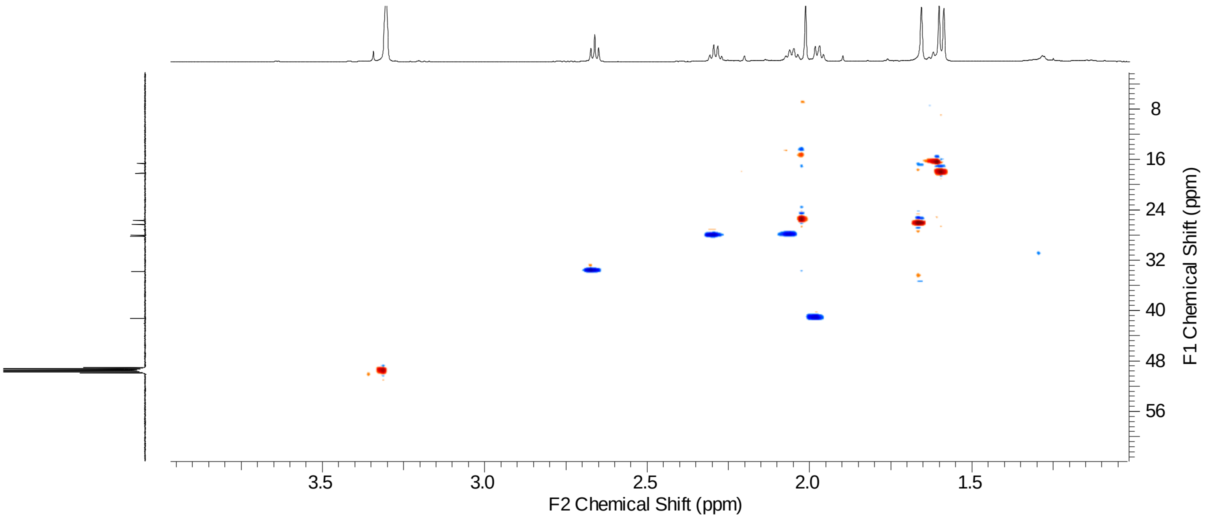

Figure S12. HMBC NMR spectrum of compound **2** in CD_3OD

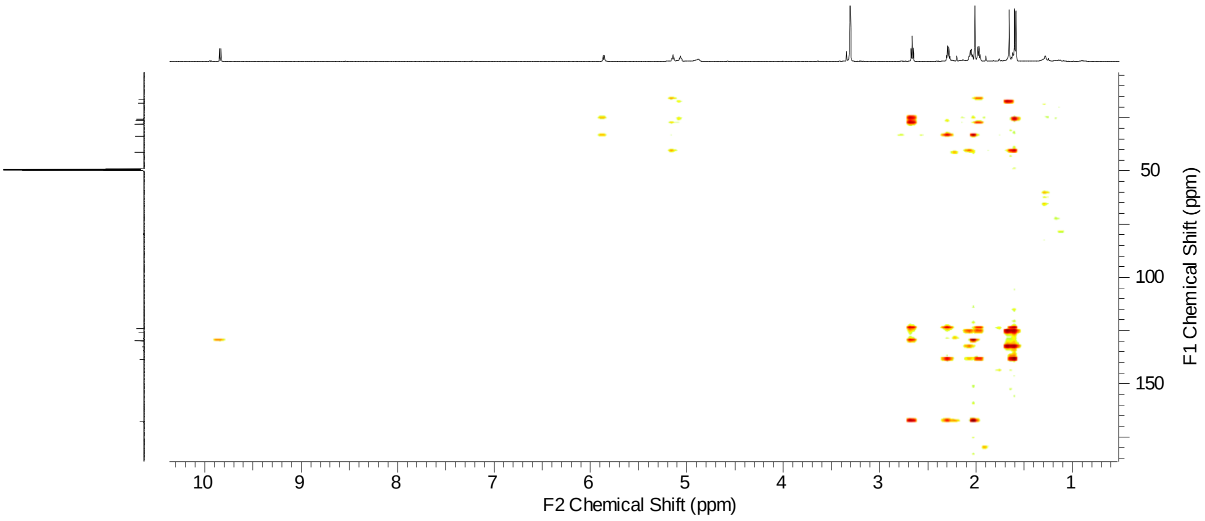

Figure S13. ^{1}H NMR spectrum of compound **3** in $CDCl_3$ (600 MHz)

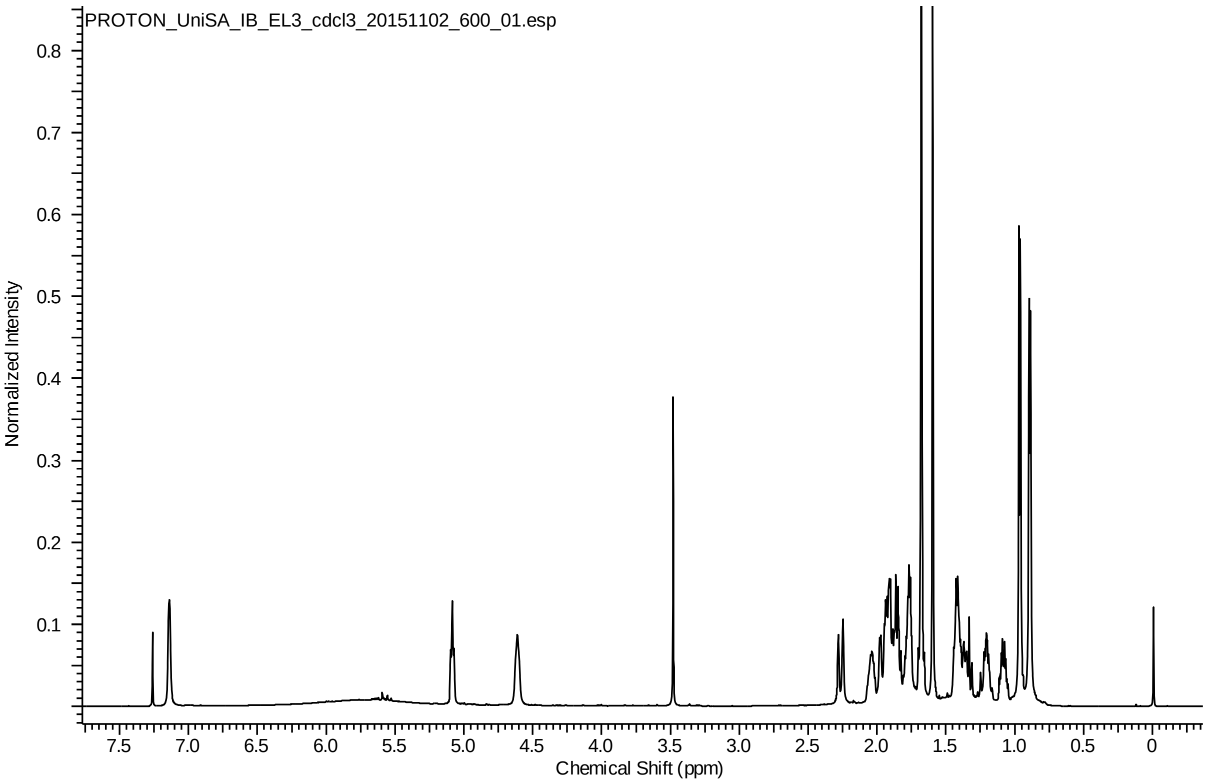

Figure S14. ^{13}C NMR spectrum of compound **3** in $CDCl_3$

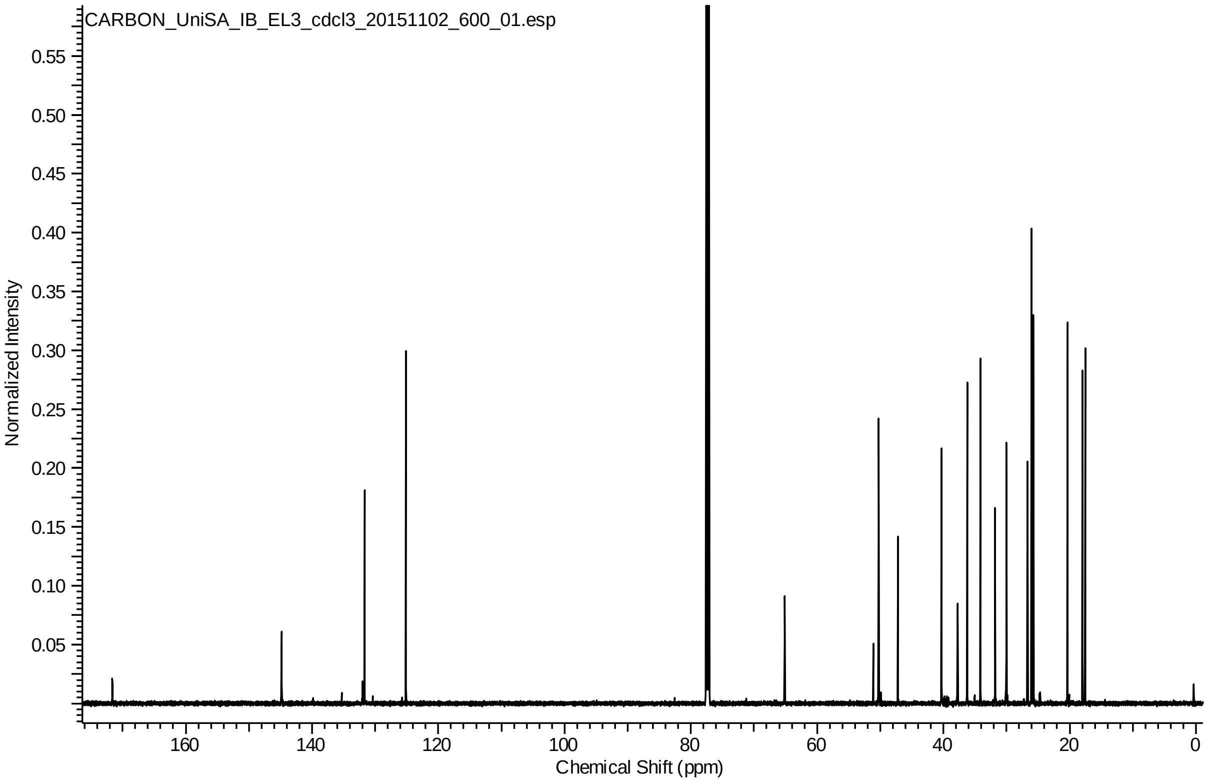

Figure S15. ^1H-^1H COSY NMR spectrum of compound **3** in $CDCl_3$

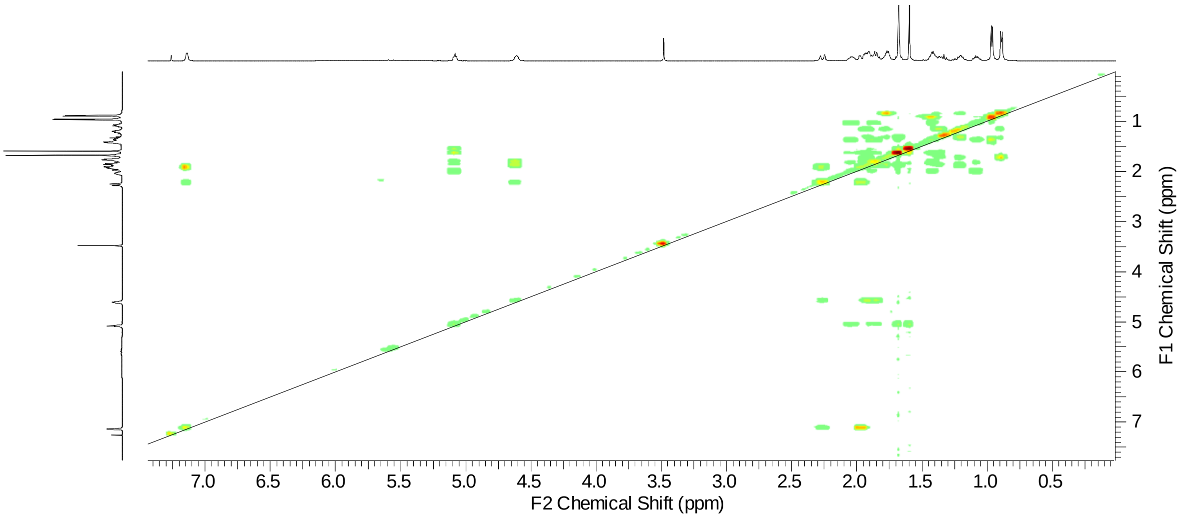

Figure S16. HSQC NMR spectrum of compound **3** in $CDCl_3$

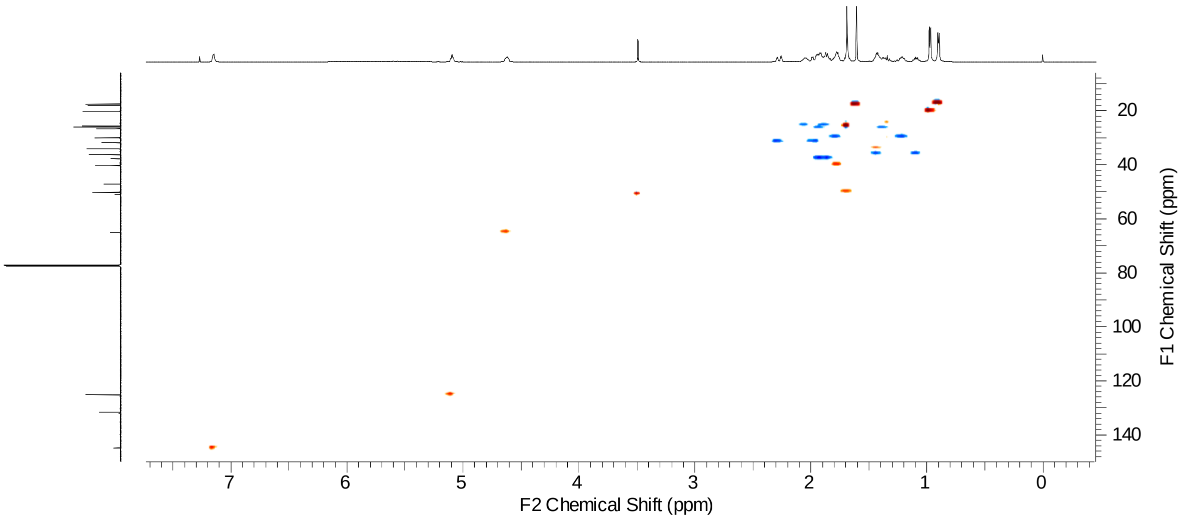

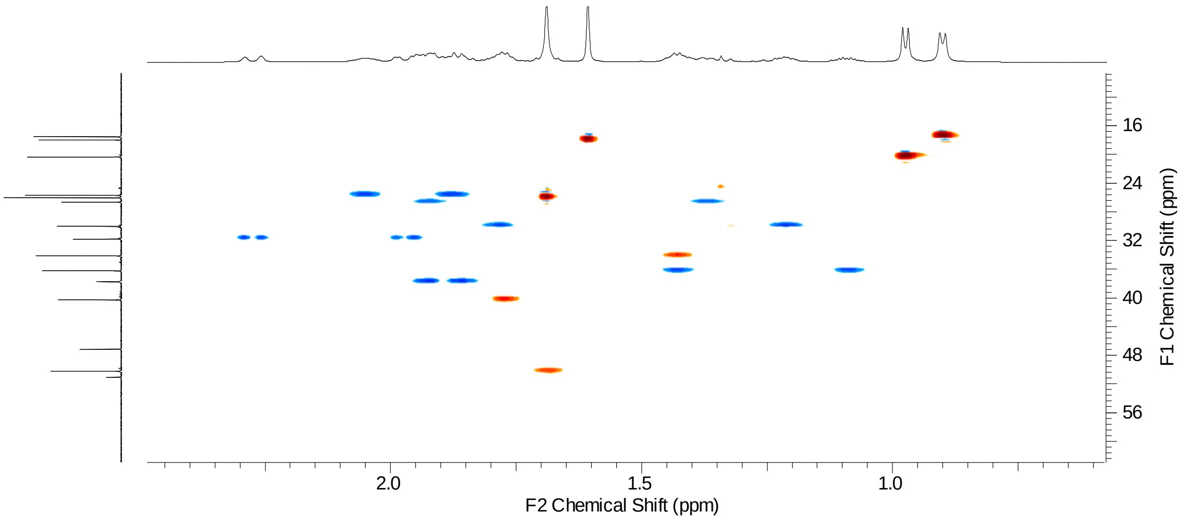

Figure S17. HMBC NMR spectrum of compound **3** in $CDCl_3$

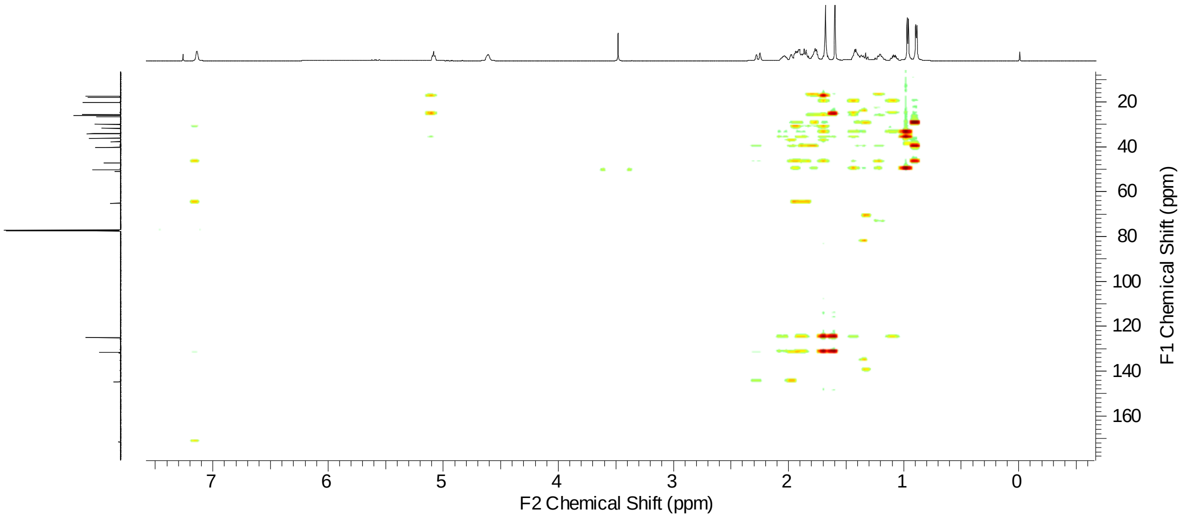

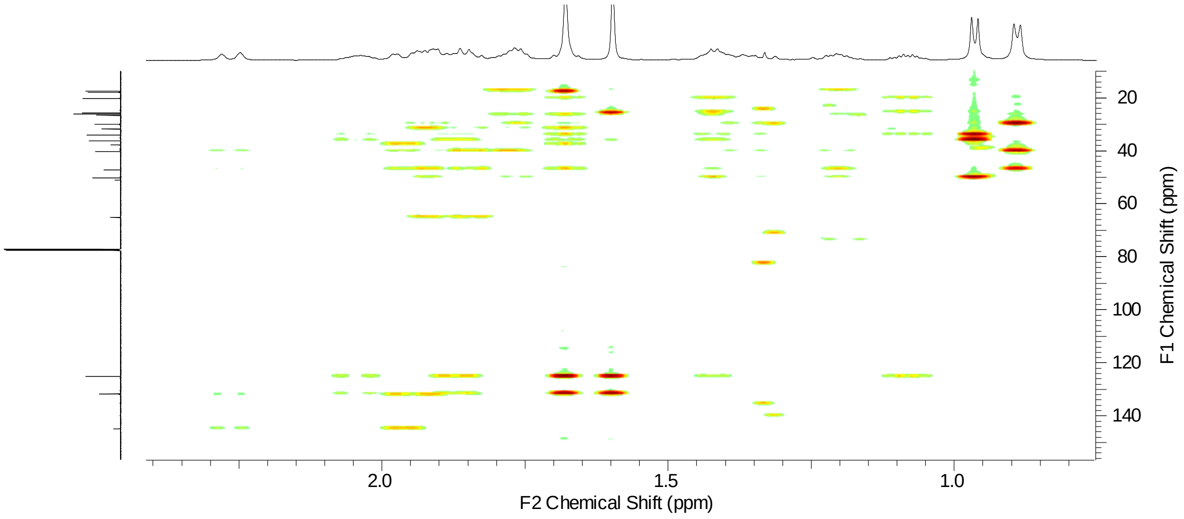

Supplement: Supplementary file 1 [file antibiotics-08-00063-s001.pdf]
